# Paper-based electrochemical immunosensor for label-free detection of multiple avian influenza virus antigens using flexible screen-printed carbon nanotube-polydimethylsiloxane electrodes

**DOI:** 10.1038/s41598-022-06101-1

**Published:** 2022-02-10

**Authors:** Daesoon Lee, Jyoti Bhardwaj, Jaesung Jang

**Affiliations:** 1 Sensors and Aerosols Laboratory, Department of Mechanical Engineering, Ulsan National Institute of Science and Technology (UNIST), Ulsan, 44919 Republic of Korea; 2 Department of Biomedical Engineering, UNIST, Ulsan, 44919 Republic of Korea; 3 Department of Urban and Environmental Engineering, UNIST, Ulsan, 44919 Republic of Korea

**Keywords:** Biotechnology, Health care, Techniques and instrumentation

## Abstract

Many studies have been conducted on measuring avian influenza viruses and their hemagglutinin (HA) antigens via electrochemical principles; most of these studies have used gold electrodes on ceramic, glass, or silicon substrates, and/or labeling for signal enhancement. Herein, we present a paper-based immunosensor for label-free measurement of multiple avian influenza virus (H5N1, H7N9, and H9N2) antigens using flexible screen-printed carbon nanotube-polydimethylsiloxane electrodes. These flexible electrodes on a paper substrate can complement the physical weakness of the paper-based sensors when wetted, without affecting flexibility. The relative standard deviation of the peak currents was 1.88% when the electrodes were repeatedly bent and unfolded twenty times with deionized water provided each cycle, showing the stability of the electrodes. For the detection of HA antigens, approximately 10-μl samples (concentration: 100 pg/ml–100 ng/ml) were needed to form the antigen–antibody complexes during 20–30 min incubation, and the immune responses were measured via differential pulse voltammetry. The limits of detections were 55.7 pg/ml (0.95 pM) for H5N1 HA, 99.6 pg/ml (1.69 pM) for H7N9 HA, and 54.0 pg/ml (0.72 pM) for H9N2 HA antigens in phosphate buffered saline, and the sensors showed good selectivity and reproducibility. Such paper-based sensors are economical, flexible, robust, and easy-to-manufacture, with the ability to detect several avian influenza viruses.

## Introduction

Influenza A viruses can cause rapid and fatal respiratory diseases in humans and some animals^1^, and they are subtyped in terms of their two surface proteins: hemagglutinin (HA) and neuraminidase^2^. In particular, many avian influenza (AI) viruses of H5 and H7 subtypes, such as H5N1 and H7N7, may have fatality rates of 100% for poultry, and AI viruses are generally classified as highly pathogenic avian influenza (HPAI) and low pathogenic avian influenza (LPAI) viruses^3^. These AI viruses can also cause fatal diseases in humans. Therefore, rapid measurement of several of these AI viruses is critical for preventing the spread of fatal respiratory diseases.

Several methods have been developed to detect AI viruses. The most typical methods are enzyme-linked immunosorbent assay (ELISA) and polymerase chain reaction (PCR)^4,5^, which are considered standard methods for virus detection because of their high accuracies and sensitivities despite drawbacks such as lengthy detection times and need for highly trained personnel. Recently, Raman spectroscopy^6^, quartz crystal microbalance^7^, and surface plasmon resonance^8^ have been used to mitigate these limitations; however, these diagnostic methods still require sophisticated equipment and appropriate training for operation^9^, thus requiring portable and user-friendly sensors that can detect AI viruses on-site to prevent the spread of these viruses effectively.

In recent years, paper-based sensors have shown great potential for on-site diagnostic kits, such as dipstick assay, lateral flow assay, vertical flow assay, and microfluidic paper-based analytical devices (μPADs)^10,11^. Paper-based sensors allow flow of fluids without external forces using only the capillary action, in addition to offering good properties for biosensors, such as flexibility, lightness, hydrophilicity, fibrous and porous structure, and a high surface-to-volume ratio, thus rendering them simple to operate as well as affordable^10,11^. The direction of fluid flow in such sensors can be controlled by printing wax or hydrophobic patterns on the paper. These paper-based sensors involve electrical, colorimetric, or electrochemical measurements^10,12,13^. Among them, the electrochemical paper-based sensors are easy to miniaturize, have low limits of detection (LODs), and can quantitatively measure the amount of analytes^14^.

To date, several studies have been presented for measuring the AI viruses or their HA antigens via electrochemical platforms (Table 1). Veerapandian et al.^15^ developed a label-free dual measurement sensor to detect the HA proteins of the influenza A viruses H1N1 and H5N1 using screen-printed carbon electrodes (SPCEs) on a ceramic substrate. The immunosensors had a detection range of 25 to 500 pM for H5 HA proteins, with an LOD of 9.4 pM^15^. A label-free sensor for detection of the HA proteins of the influenza virus H5N1 was also developed using a gold electrode on a silicon oxide wafer and a 3-way-junction DNA; it had a detection range of 1 pM to 100 nM, with an LOD of 1 pM^16^. As can be seen from Table 1, most of these previous studies using electrochemical platforms have incorporated gold electrodes or SPCEs on ceramic, glass, polymer, or silicon substrates but not on paper, and/or they used labeling for signal enhancement.

**Table 1:** Electrochemical immunosensors for detection of avian influenza viruses (H5N1, H7N9, and H9N2), their hemagglutinin (HA) proteins, or peptides derived from the hemagglutinin proteins.

Probe	Measuring technique	Target	Detection range	Labeling	Limit of detection	Electrode	Substrate	References
**H5N1 HA or virus**								
Antibody	EIS	H5N1 virus	3–7 (log EID50/ml)	RBC	3 (log EID50/ml)	Gold (IDA)	Glass	^23^
Antibody	Impedance measurement	H5N1 virus	1–5 (log EID50/ml)	RBC	3 (log EID50/ml)	Gold (IDA)	NA	^24^
Antibody fragment	EIS	Peptide from HA (H5N1)	4–20 pg/ml	Label free	2.2 pg/ml	Gold	Polymer	^25^
Antibody	EIS	H5N1 virus	0.25–16 (HAU/50 µl)	Label free	0.5 (HAU/50 µl)	Gold (IDA)	Glass	^26^
Aptamer	DPV	HA (H5N1)	0.1–10 pM	ALP-Ab	0.1 pM	AuNP-SPCE	NA	^27^
Aptamer	Impedance measurement	H5N1 virus	0.125–16 (HAU/50 µl)	Gold nanoparticle	0.25 (HAU/50 µl)	Gold (IDA)	NA	^28^
Antibody	DPV	HA (H5N1)	25–500 pM	Label free	9.4 pM	SPCE	Ceramic	^15^
Antibody fragment	DPV, EIS	Peptide from HA (H5N1)	4–20 pg/ml 1–8 pg/ml	Label free	0.6 pg/ml 0.9 pg/ml	Gold	Polymer Ceramic	^29^
Antibody	CV	H5N1 virus	0.0025–0.16 HAU	MNP	0.0022(HAU/6 µl)	Gold	NA	^30^
3 way-junction DNA	CV	HA (H5N1)	1 pM–100 nM	Label free	1 pM	Gold	SiO2	^16^
**H7N9 HA or virus**								
Antibody	LSV	H7N9 virus	10 pg/ml–20 ng/ml	bi-MBs	6.8 pg/ml	Glassy carbon	NA	^31^
Antibody	LSV	HA (H7N9)	1.6 pg/ml–1.6 ng/ml	PAb-AgNPs-G	1.6 pg/ml	Gold	NA	^32^
Antibody	Amperometry	H1N1, H5N1, & H7N9 viruses	1 pg/ml–10 ng/ml	HRP-Abs	1 pg/ml	Gold	Glass	^33^
Antibody	LSV	H7N9 virus	0.01–1.5 pg/ml	Fluorescence MNP	7.8 fg/ml	ITO	Glass	^34^
**H9N2 HA or virus**								
Antibody	DPV	H9N2 virus	1 ng/ml–2 µg/ml	GOD-A	1 (ng/ml)	Gold	Magnet	^35^
Antibody	Chronoamperometry	H9N2 virus	8–128 HAU	AuNP-fetuin	8 (HAU)	SPCE	Ceramic	^36^
Antibody	DPV	HA (H5N1, H7N9, and H9N2)	100 pg/ml–100 ng/ml (1.7 pM–1.7 nM)	Label free	55.7 (pg/ml) for H5N1 HA (0.95 pM) 99.6 (pg/ml) for H7N9 HA (1.69 pM) 54.0 (pg/ml) for H9N2 HA (0.72 pM)	SPCE	Paper	Present study

*IDA* interdigitated electrode array, *SPCE* screen-printed carbon electrode, *EID* egg infectious dose, *HAU* hemagglutinin units, *RBC* red blood cell, *bi-MBs* bifunctional magnetic beads, *HRP-Abs* horseradish peroxidase antibodies, *GOD-A* glucose oxidase conjugated avidin D, *ALP* alkaline phosphatase, *MNP* magnetic nanoparticle, *PAb-AgNPs-G* polyclonal antibody/silver nanoparticle/graphene conjugate, *ITO* indium titanium oxide, *EIS* electrochemical impedance spectroscopy, *DPV* differential pulse voltammetry, *CV* cyclic voltammetry, *LSV* linear sweep voltammetry, *NA* not available.

**Figure 1:**
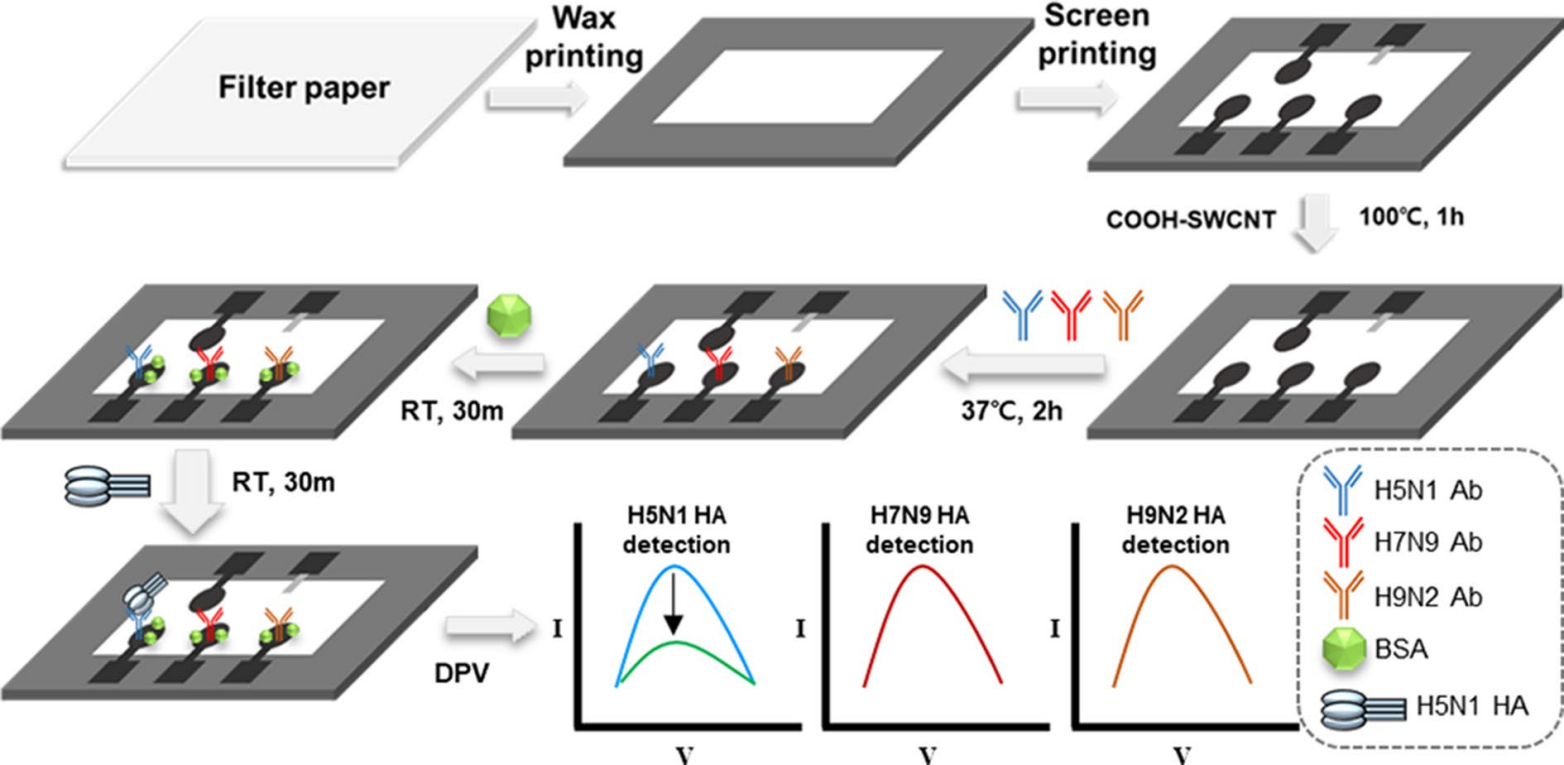
Schematic of the proposed paper-based electrochemical immunosensor for detection of three different avian influenza virus antigens. Here, H5N1 HA alone is present on the sensor for the purpose of illustration. RTs stand for room temperature, ~ 25 ℃.

Herein, we present a label-free electrochemical paper-based sensor for detecting multiple AI virus antigens (Fig. 1), and this study would be the first to quantity multiple AI virus antigens using an electrochemical paper-based sensor. Generally, these AI viruses must be handled in biosafety level 3 or higher laboratories^17^; hence, their virus antigens are safer to use, especially in the event that aerosolization of the AI viruses may be needed^18–20^. These sensors have also shown cost-effective and simple fabrication processes, such as hydrophobic patterning using wax printing, screen-printing of the electrodes, and drop-casting of COOH-functionalized single-walled carbon nanotubes (COOH-CNTs) for antibody immobilization.

Moreover, continuous monitoring of airborne biological agents over time may require roll-to-roll sensors on flexible substrates such as paper^21^ and polymers. A paper substrate can absorb and keep analytes in sample liquids without a container. In contrast, a polymeric substrate is usually hydrophobic; hence, liquids on the substrate, when dropped, can move freely unless they are contained. In order to use the paper-based sensors for the purposes, robust and flexible electrodes are needed to obtain stable electrochemical signals since erroneous measurements can be made due to repeated bending and unfolding of paper substrate. In the present study, multi-walled (MW) CNTs and polydimethylsiloxane (PDMS) were mixed to produce a MWCNT-PDMS paste for screen-printing flexible electrodes on paper substrate. The CNT-PDMS composite is physically robust and flexible, with good reproducible electrical resistance^22^. This composite has been generally used for pressure or tactile sensors, but rarely used for electrochemical biosensors despite the advantages for the paper-based sensors. Three working electrodes were manufactured, and antibodies for three AI viruses (H5N1, H7N9, and H9N2) were immobilized on the COOH-functionalized CNTs via 1-ethyl-3-(3-dimethylaminopropyl) carbodiimide (EDC) and N-hydroxysuccinimide (NHS). The bending test, sensitivity, selectivity, and reproducibility of these sensors were measured and discussed.

## Results and discussion

### Characteristics of the MWCNT-PDMS electrodes

**Figure 2:**
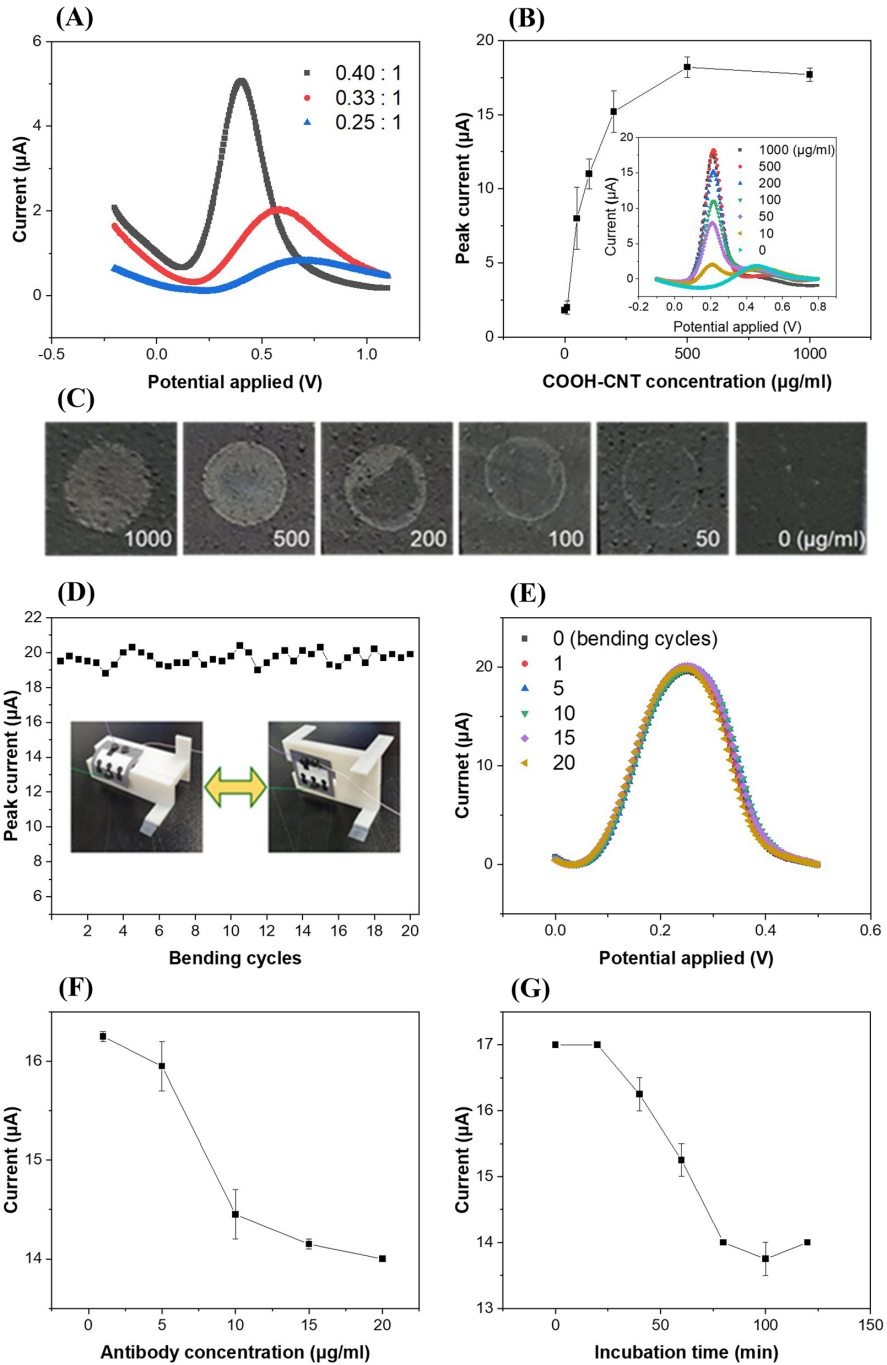
**(A)** Differential pulse voltammograms of screen-printed MWCNT-PDMS electrodes with different mixing ratios of MWCNT and PDMS. **(B)** Peak currents measured for various concentrations of COOH-CNTs. The inset shows the differential pulse voltammograms for each deposition concentration of COOH-CNT on the working electrode. **(C)** Photograph of the working electrodes after placing 2 µl of dimethylformamide containing carboxyl-functionalized single-walled carbon nanotubes (COOH-CNTs) for different concentrations ranging from 0 to 1000 µg/ml, followed by curing in an oven at 100 °C. **(D)** Peak currents of the COOH-CNT/MWCNT-PDMS electrodes measured with repeating bending and unfolding for 20 cycles. The inset shows the photographs of the bending test setup. **(E)** Differential pulse voltammograms of the COOH-CNT/MWCNT-PDMS electrodes at 1, 5, 10, 15, and 20 bending cycles. **(F)** Peak currents measured for different antibody concentrations on the working electrodes (incubation time = 120 min, concentration: 1, 5, 10, 15, and 20 µg/ml). **(G)** Peak currents measured for antibody incubation times (concentration: 10 µg/ml, t = 0, 20, 40, 60, 80, 100, and 120 min, temperature = 37 °C).

The peak currents were measured by differential pulse voltammetry (DPV) as a function of the weight ratio of the MWCNTs to PDMS in the MWCNT-PDMS electrodes; the peak current was observed to increase with increasing ratio of MWCNTs in the MWCNT-PDMS composite (Fig. 2A). In the case of 0.25:1, the DPV peak current was 0.8 µA at 0.72 V. When the MWCNT proportion was increased to 0.40:1, the peak current increased to 5.0 µA at 0.45 V; the main reason for this increase in peak current and decrease in peak potential with decreasing PDMS proportion can be due to the dielectric properties of PDMS^37^, which cause changes in the electron transfer kinetics between the electrode interface and electrolyte^38,39^. When the composite ratio was higher than 0.40:1, the composite was not of a paste form available for screen-printing.

### Working electrode characterization and bending test

We examined the effects of the concentration of COOH-CNTs, and the concentration and incubation time of antibodies on the COOH-CNTs modified electrodes. Figure 2B shows the peak currents measured after immobilizing 2 μl of varying concentrations (0–1000 µg/ml) of COOH-CNTs on the working electrodes and baking them in an oven at 100 ℃. Increases in peak current and shifts in peak potential (from 0.45 to 0.2 V) were observed after immobilization of COOH-CNTs on the MWCNT-PDMS electrode. The reason for this can be attributed to high conductivity of COOH-CNT that enhanced the electron transfer between the working electrodes and electrolyte^38,39^, also implying that the electrochemical surface characteristics of the MWCNT-PDMS electrode changed because of COOH-CNT deposition. The peak current increased with increase in the COOH-CNTs concentration, and saturated at 18.25 μA for a concentration of 500 μg/ml, where a peak potential of 0.45 V was no longer observed. This means that COOH-CNTs sufficiently covered the PDMS-MWCNT electrodes in terms of electrochemical measurement.

For visual observation, images of the working electrodes were also captured with a smartphone camera after various concentrations of COOH-CNTs were immobilized on the working electrodes and dried in an oven at 100 ℃ (Fig. 2C). The bright gray color in the images indicate the deposition of COOH-CNTs on the electrode surfaces. The coffee ring effect^40^ was observed after deposition of COOH-CNTs in the concentration range from 50 to 200 µg/ml, where the black color showed negligible deposition of COOH-CNTs on the surface, demonstrating that these concentrations of COOH-CNTs were not enough to cover the whole electrode surfaces. However, the electrodes were fully covered (bright gray color) with COOH-CNTs after deposition of COOH-CNTs in a concentration range from 500 to 1000 µg/ml, which indicates that 500 µg/ml is the optimal concentration for complete coverage without clumps on the edges. This CNT concentration is comparable to that observed in another study, where the CNT concentration was around 200 μg/ml when using the drop-casting method^41^.

To assure the robustness and flexibility of these paper-based sensors, the peak currents of these electrodes were measured using DPV by repeatedly bending and unfolding twenty times (Fig. 2D)^42^. The peak currents varied from 18.8 μA to 20.4 μA, where the relative standard deviation (RSD) was 1.88%, which was lower than that (3.09%) observed in the reproducibility tests. This shows that the COOH-CNT/MWCNT-PDMS electrodes were minimally affected by mechanical bending, while the electrochemical sensors were continuously wetted, and the bent COOH-CNT/MWCNT-PDMS electrodes exhibited very good stability (Fig. 2E). Although the bending test is one of the major considerations for a flexible biosensor, paper-based electrochemical biosensors showing a bending test have rarely been presented to date^42,43^. This may be because such sensors are usually developed as disposable devices, and continuous monitoring of airborne viruses using disposable and flexible immunosensors, which may require that the sensors should be bent and unfolded^21^, has rarely been reported despite its high impact^44,45^. Moreover, the proposed MWCNT-PDMS electrodes on a paper substrate have the potential for flexible electrochemical biosensor applications, such as polymer-based roll-to-roll printed biosensor patches^46^.

The peak currents were also measured after immobilizing 10 μl of antibody solution on the modified MWCNT-PDMS electrodes in a concentration range from 1 to 20 μg/ml (Fig. 2F). The peak current decreased with increase in the concentration of antibodies up to 10 μg/ml, which might be due to insulating behavior of antibodies. When the antibody concentration was greater than 10 μg/ml, the peak current became saturated, which indicated that most of the free and activated carboxyl groups on the working electrodes bound with the antibodies. Optimization was also performed for incubation time of the antibodies. Ten microliters of antibodies in PBS (concentration: 10 μg/ml) was dropped on the modified MWCNT-PDMS electrodes and incubated in an oven at 37 ℃ up to 120 min to measure changes in the electrochemical properties with time (Fig. 2G). The peak current decreased with incubation time and converged at durations over 80 min.

### Electrochemical characterization of the functionalized working electrodes

**Figure 3:**
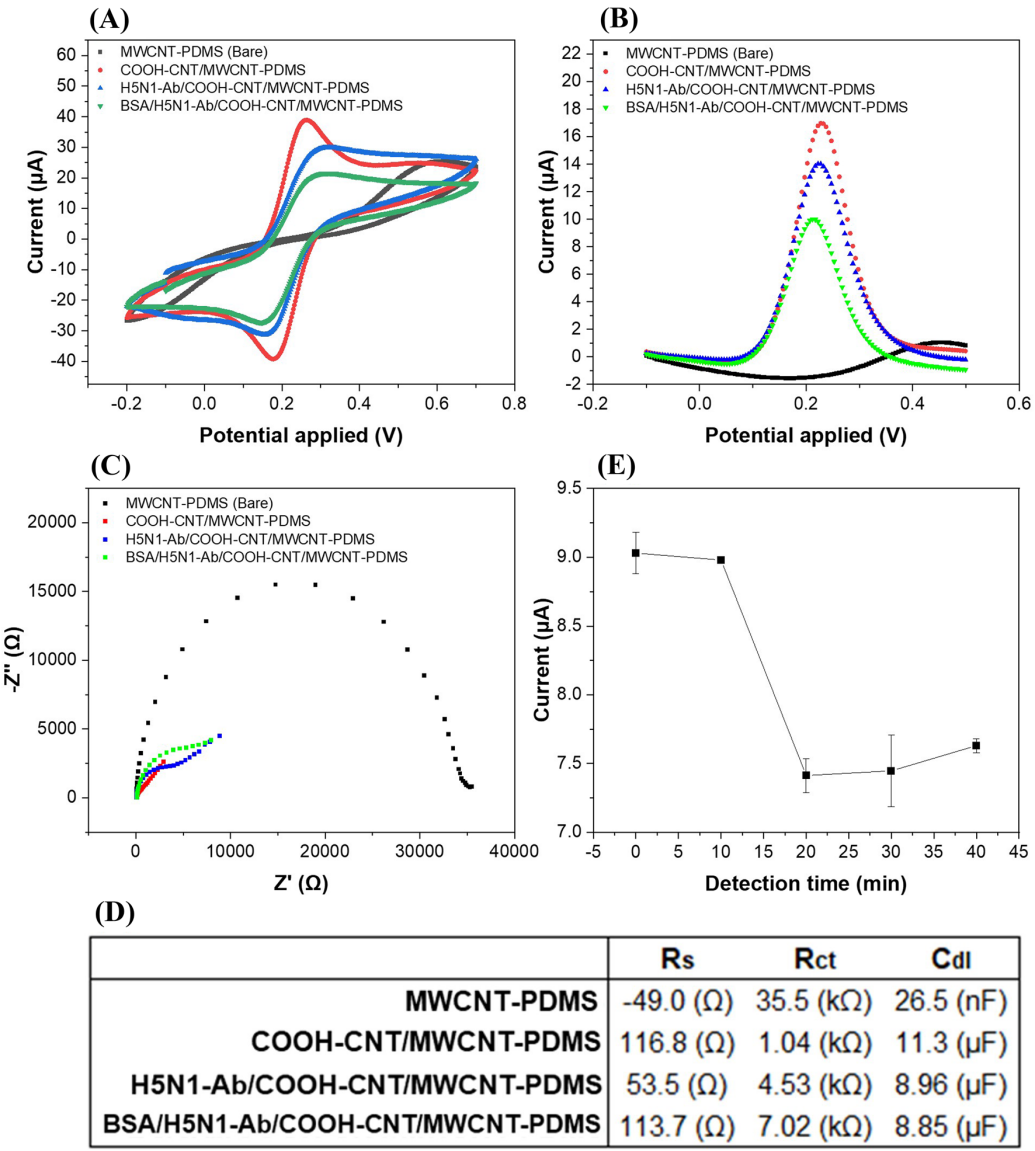
**(A)** Cyclic voltammograms (scan rate: 50 mV/s), **(B)** differential pulse voltammograms, and **(C)** electrochemical impedance spectra of the MWCNT-PDMS, COOH-CNT/MWCNT-PDMS, H5N1-Ab/COOH-CNT/MWCNT-PDMS, and BSA/H5N1-Ab/COOH-CNT/MWCNT-PDMS electrodes. The inset shows the equivalent circuit for EIS measurement. **(D)** Measured values of the solution resistance (R_s_), charge transfer resistance (R_ct_) and double layer capacitance (C_dl_) in the MWCNT-PDMS, COOH-CNT/MWCNT-PDMS, H5N1-Ab/COOH-CNT/MWCNT-PDMS, and BSA/H5N1-Ab/COOH-CNT/MWCNT-PDMS electrodes. **(E)** The DPV peak currents measured for different incubation times for 10 µl of H5N1 HA detection (0, 10, 20, 30, and 40 min, concentration: 100 pg/ml, temperature = 25 °C). The error bars represent the standard deviations of three independent measurements.

Electrochemical properties of the functionalized working electrodes with the deposition process were measured using DPV, CV, and EIS. Figure 3A shows the cyclic voltammograms for the MWCNT-PDMS (bare electrode), COOH-CNT/MWCNT-PDMS, Ab/COOH-CNT/MWCNT-PDMS, and BSA/Ab/COOH-CNT/MWCNT-PDMS electrodes. We observed a peak current of 38.9 μA at 0.2 V after deposition of COOH-CNTs on the bare electrodes, which had no CV peaks at 0.2 V. This was due to conductive behavior of COOH-CNTs via increasing the surface area and the electron transfer rate between the electrodes and electrolyte. The peak current decreased up to 29.9 µA after immobilizing antibodies on the modified electrode surface, which may be due to insulating behavior of antibodies. The peak current further decreased to 21.5 μA after immobilization of BSA. Large size and insulation properties of proteins such as BSA and antibodies decrease the electron transfer between electrodes and electrolyte^11^. DPV was also used for the same processes (Fig. 3B) and similar behaviors were observed. The peak currents were 16.9 μA after COOH-CNT deposition, 14.0 μA after antibody deposition, and 9.8 μA after BSA deposition.

The impedance spectroscopy was performed to measure the interfacial changes after each modification of the electrode surface (Fig. 3C) at an electric potential of 0.1 V and an amplitude of 10 mV in a frequency range from 0.1 Hz to 100 kHz. The inset shows the equivalent circuit, where R_ct,_ R_s,_ C_dl_ and W represent the charge transfer resistance, solution resistance, double layer capacitance, and Warburg effect, respectively (Fig. 3D). The charge transfer resistance (R_ct_) of 35.5 kΩ for the bare electrodes was significantly reduced to 1.04 kΩ after COOH-CNT deposition. The R_ct_ of the Ab/COOH-CNT/MWCNT-PDMS increased to 4.53 kΩ due to decrease in electron transfer after antibody immobilization, which hinders electron transfer. The R_ct_ value of the BSA/Ab/COOH-CNT/MWCNT-PDMS electrodes were increased to 7.0 kΩ, indicating that the BSA covered nonspecific sites of the Ab/COOH-CNT/MWCNT-PDMS electrodes and obstructed the electron transfer.

The effect of incubation periods was measured to determine the optimal detection time for efficient binding of HA proteins on the antibodies-modified electrode surfaces. Figure 3E shows the peak currents after incubating H5N1 HA on the BSA/H5N1-Ab/COOH-CNT/MWCNT-PDMS electrodes for various time periods (10–40 min). Incubation of H5N1 HA on the sensors resulted in an antigen–antibody reaction and hence a change in the DPV peak current. The peak current saturated at 20–30 min, resulting in the maximum antigen–antibody binding.

### Differential pulse voltammetry for hemagglutinin antigen detection

**Figure 4:**
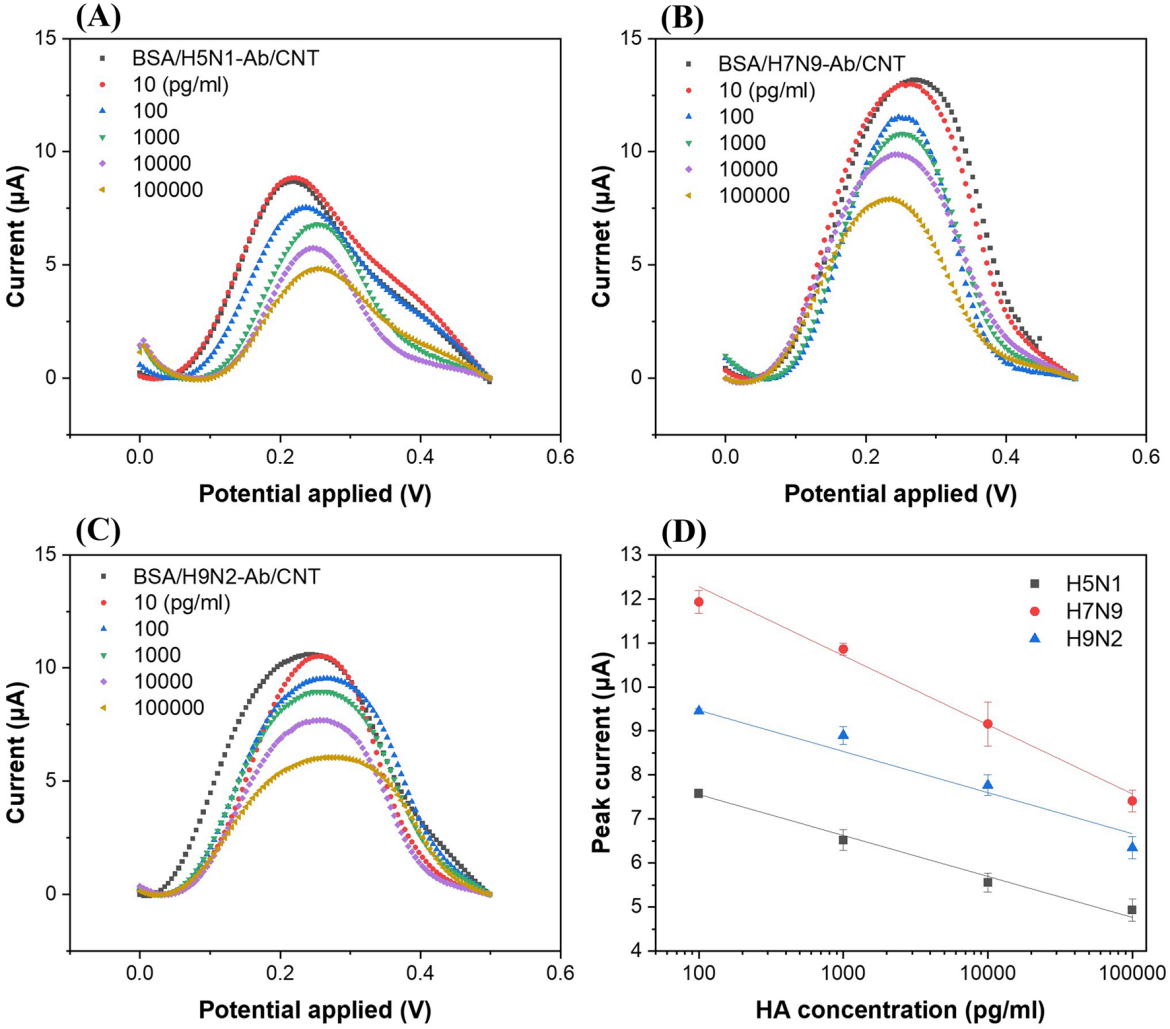
Differential pulse voltammograms of different concentrations of hemagglutinin antigens of the influenza virus **(A)** H5N1, **(B)** H7N9, and **(C)** H9N2 subtypes. **(D)** Calibration graphs of hemagglutinin antigens of influenza virus H5N1, H7N9, and H9N2 for the detection range from 100 pg/ml to 100 ng/ml. The error bars represent the standard deviations of three independent measurements.

Three different HAs of the AI viruses (H5N1, H7N9, and H9N2) were detected using DPV (Fig. 4). Ten microliters of the HA solutions (in PBS) of different concentrations (10 pg/ml–100 ng/ml or 0.00017 nM–1.7 nM) were incubated on the BSA/Ab/COOH-CNT/MWCNT-PDMS electrodes for 30 min and then washed with PBS, followed by measurements with DPV. Figure 4A–C showed decreases in peak current with increases in concentration of HA proteins of H5N1, H7N9, and H9N2, respectively, in the range of 10 pg/ml–100 ng/ml. The previous studies for HA detection using electrochemical sensors were conducted in a similar concentration range (Table 1). The injected HA bound with the antibodies immobilized on the working electrodes, and peak currents decreased with increasing HA concentrations due to the electrical insulation property of HA proteins.

Figure 4D shows the log-linear relationships between the DPV peak currents and the three HA (H5N1, H7N9, and H9N2) concentrations. The LODs were computed as 55.7 pg/ml (0.95 pM) for H5N1 HA, 99.6 pg/ml (1.69 pM) for H7N9 HA, and 54.0 pg/ml (0.72 pM) for H9N2 HA, based on the three signal-to-noise ratios. Compared to other works on electrochemical detection of influenza HAs (Table 1), where the LODs were 9.4 pM^15^ and 1 pM^16^ for H5N1 HA proteins, present sensors showed similar or enhanced detection limits for H5N1 HA proteins. Han et al.^33^ reported a microfluidic immunosensor for simultaneous detection of H1N1, H5N1, and H7N9 viruses using ZnO nano-rods and HRP conjugated antibodies for signal enhancements with LOD of 1 pg/ml; however, the detection time was long (more than 2 h) because they used 1 h for incubation time of antigens and then another 1 h incubation of conjugated antibodies, followed by washing and detection^33^. The present sensors showed rapid (20–30 min) detection of multiple AI antigens using a simple, cost-effective and flexible paper-based electrochemical immunosensor. Moreover, there are no other studies reported on multiple detection of H5N1, H7N9, and H9N2 HA antigens in a single electrochemical paper-based device.

### Multiple detection test

**Figure 5:**
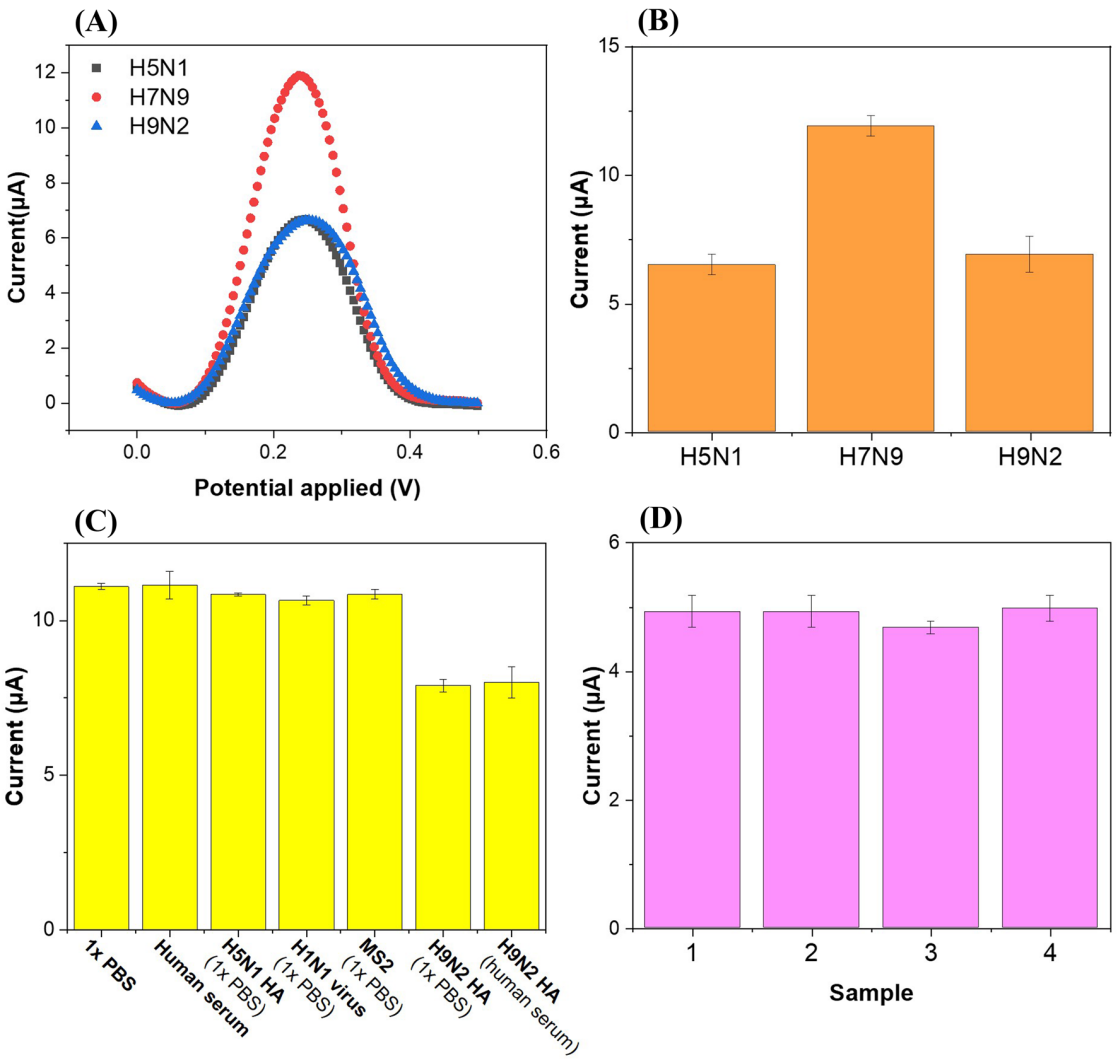
**(A)** Differential pulse voltammograms and **(B)** peak currents of three BSA/Ab/COOH-CNT/CNT-PDMS electrodes of the proposed immunosensors after 10 $\mathrm{\mu l}$ of 1 × PBS containing 10 ng/ml of H5N1 HA and 10 ng/ml of H9N2 HA was injected and incubated at room temperature for 30 min. **(C)** Selectivity of the immunosensors (H9N2-Ab functionalized electrodes) for influenza virus H9N2 hemagglutinin antigen detection. The test included 1 × PBS only, ten-fold diluted human serum only, non-targets (H5N1 HA, H1N1 virus, and MS2 bacteriophage) in 1 × PBS, and targets in 1 × PBS or ten-fold diluted human serum. **(D)** Reproducibility of the immunosensors (H5N1-Ab functionalized electrodes) using H5N1 HA antigen (concentration: 100 ng/ml). The error bars represent the standard deviations of three independent measurements.

To demonstrate the detection capabilities of the sensors for multiple targets, we tested one case in this study although there are many combinations of 3 three different HAs and several antigen concentrations. A 10-μl PBS containing H5N1 HA (10 ng/ml) and H9N2 HA (10 ng/ml), where no H7N9 HAs were included, was injected into each of the three working electrodes (BSA/H5N1-Ab/COOH-CNT/MWCNT-PDMS, BSA/H7N9-Ab/COOH-CNT/MWCNT-PDMS, and BSA/H9N2-Ab/COOH-CNT/MWCNT-PDMS electrodes) and incubated for 30 min at room temperature before measuring the electrochemical changes on each working electrode sequentially (Fig. 5A,B). The peak currents measured after the HA sample injection were 6.6 μA and 6.9 μA for H5N1-Ab and H9N2-Ab functionalized electrodes, respectively, whereas 11.9 μA was obtained for H7N9-Ab functionalized electrodes, similar to those of the negative controls. The peak currents of the single HA tests showed 5.8 μA (*p* = 0.19) at 10 ng/ml of H5N1 HA and 7.6 μA (*p* = 0.84) at 10 ng/ml of H9N2 HA (Fig. 4D), demonstrating that both single and multiple analyte detections were not significantly different for the 95% confidence intervals. Based on the observations, these immunosensors can quantify the concentrations of multiple HA proteins and showed negligible interference between target and non-target HAs in the samples.

### Selectivity and reproducibility tests

For selectivity assay, different viruses (influenza A H1N1 whole viruses and MS2 bacteriophages) and HA proteins in PBS and human serum were used (Fig. 5C). The human serum was ten-fold diluted with PBS. H9N2-Ab functionalized electrodes were incubated with 10 μl of either H5N1 HA, H1N1 viruses, H9N2 HA (target), or MS2 bacteriophages for 30 min and washed with PBS before DPV measurements. The negative controls (PBS and human serum) were obtained by measuring the peak current of the H9N2-Ab functionalized electrodes after incubation of PBS and human serum alone, respectively. The concentration used in the experiments was 10 ng/ml for HA and 10^4^ plaque forming units (pfu)/ml for viruses and bacteriophages. Only the DPV peak currents for H9N2 HA decreased, unlike those for the other non-targets and the negative controls. This implies that the immunosensor shows good selectivity.

The reproducibility tests of these sensors were conducted (Fig. 5D). All H5N1-Ab functionalized electrodes were given the same concentration of H5N1 HA (100 ng/ml), incubated for 30 min, washed with PBS, and measured by DPV. The peak currents did not differ significantly, and the RSD was 3.09%, showing good reproducibility for label-free detection.

## Conclusion

In this study, a paper-based electrochemical immunosensor was presented for quantifying multiple AI virus antigens in a label-free manner using flexible screen-printed CNT-PDMS electrodes. Three working electrodes were constructed within a single sensor, and three different antibodies were immobilized on each working electrode; therefore, three different AI HA proteins can be measured separately. Screen-printed carbon electrodes were made by using MWCNT-PDMS paste to protect the paper substrate that might be fragile to unavoidable liquid processes as well as to provide stability and flexibility (RSD: 1.88%) under 20 cycles of bending and unfolding. COOH-CNTs were then attached to the working electrodes using drop casting. H5N1, H7N9, and H9N2 HA antigens were detected at concentrations from 100 pg/ml to 100 ng/ml, and the LODs of the sensors were 55.7 pg/ml (0.95 pM) for H5N1 HA, 99.6 pg/ml (1.69 pM) for H7N9 HA, and 54.0 pg/ml (0.72 pM) for H9N2 HA antigens, showing good selectivity and reproducibility (RSD: 3.09%) as well. Thus, this paper-based electrochemical immunosensor has high potential for field detection of AI viruses owing to its low cost, high sensitivity, simple measurement with a portable potentiostat, as well as rapid measurement time (20–30 min).

## Materials and methods

### Materials

Whatman filter paper (grade 4, pore size: 20–25 µm) was purchased from GE Healthcare Inc. (USA). The MWCNTs were purchased from Graphene Supermarket Inc. (USA). An adhesive polyester film (thickness: 100 μm) was obtained from Fancylobby (South Korea); PDMS was purchased from K1 Solution Co., Ltd. (South Korea). Further, Ag/AgCl ink was obtained from ALS Co., Ltd. (Japan). COOH-CNTs (> 90% carbon basis, diameter: 4–5 nm, length: 0.5–1.5 µm, 1.0–3.0 atom% carboxylic acid), EDC, NHS, human serum, and bovine serum albumin (BSA) were obtained from Sigma-Aldrich (USA). One mole of 2-(N-morpholino)ethanesulfonic acid (MES) buffer was obtained from Tech & Innovation (South Korea), dimethylformamide (DMF) was purchased from Daejung Chemicals & Metals Co., Ltd. (South Korea), and phosphate-buffered saline (1 × PBS, pH 7.4) was obtained from Thermo Fisher Scientific Inc. (USA); influenza A H5N1 HA polyclonal antibody (pAb) (Rabbit pAb, 11048-RP02), influenza A H7N9 HA pAb (Rabbit pAb, 40103-RP01), influenza A H9N2 HA pAb (Rabbit pAb, 11229-RP02), influenza A H5N1 HA proteins (A/Anhui/1/2005, 11048-V08H4), influenza A H7N9 HA proteins (A/Shanghai/1/2013, 40104-V08H), and influenza A H9N2 HA proteins (A/Hong Kong/35820/2009, 40174-V08B) were purchased from Sino Biological Inc. (China). MS2 bacteriophages (ATCC 15597-b1) and influenza A virus H1N1 (KBPV-VR-76) were obtained from the Korean Bank for Pathogenic Viruses (South Korea).

### Sensor electrode fabrication

The wax pattern showing the sensing areas of the sensors was designed using Auto-CAD and printed on a Whatman filter paper (35 mm × 35 mm) using a solid wax printer (ColorQube 8570, Xerox). The paper was placed in an oven at 100 ℃ for 10 min to allow the wax to melt into the paper and then removed from the oven for hardening.

The electrochemical measurement was based on the three-electrode system, and the electrodes were manufactured by screen printing. The stencil mask design consisted of three working electrodes, one counter electrode, and one reference electrode. The design was machined over a one-sided adhesive polyester film (height: 100 μm) using a laser cutter (Micro Laser Cutter C40, Coryart), and the adhesive film was attached to the waxed paper. The three working electrodes and counter electrode were fabricated from an MWCNT-PDMS paste, which was a mixture of MWCNT and PDMS to form a screen-printable dough. Because the size and surface area of the CNTs vary depending on the manufacturer, their proportion may change^22^. The MWCNT-PDMS dough was printed on the stencil mask and baked at 70 ℃ for 2 h, followed by cooling at room temperature (25 ℃) and removal of the stencil mask. The sensing areas of the electrodes were located within the hydrophilic non-waxed areas, and the hydrophobic areas, which were not in contact with the electrolytes, were connected to the electrochemical analyzer. For the reference electrode, the MWCNT-PDMS dough was printed only in the hydrophobic areas, and Ag/AgCl ink was printed in the hydrophilic area and baked at 70 ℃ for 30 min. The circular areas of the working electrodes and counter electrode were 12.6 mm^2^ (diameter: 4 mm), and the area of the reference electrode within the hydrophilic area was 3.8 mm^2^.

### Sensor electrode functionalization

COOH-CNTs were deposited onto the three working electrodes to increase the conductivity of the electrodes^47^. COOH-CNTs were dissolved in DMF at a concentration of 500 μg/ml and sonicated for 2 h. The COOH-CNT supernatant was then collected by centrifuging at 4000 rpm for 1 h^48^. A 2 μl of the COOH-CNT solution was placed on each of the working electrodes and baked in an oven at 100 ℃ for 1 h, followed by washing with distilled water and drying with nitrogen gas.

EDC/NHS was used as a crosslinker for covalent bonding of COOH-CNTs with the antibodies via amide bonds. The mixture of EDC (10 μl, 15 mM in 1 M MES buffer) and NHS (10 μl, 30 mM in 1 M MES buffer) was incubated on the working electrodes at room temperature for 30 min to activate the carboxyl groups of the COOH-CNTs. The electrodes were then washed with PBS to remove any residual EDC, NHS, and MES buffer. For each of the working electrodes, approximately 10 μl of the antibodies (concentration: 10 μg/ml) specific to the HA proteins of influenza viruses (either H5N1, H7N9, or H9N2) were injected separately, incubated at 37 ℃ for 2 h, washed with PBS, and then dried with nitrogen gas. Ten microliters of BSA solution (10 mg/ml in PBS) was then placed on each working electrode and incubated at room temperature for 30 min to prevent nonspecific binding, followed by washing with PBS (Fig. 1).

### Bending test

We also explored the possibility that the COOH-CNT/MWCNT-PDMS electrodes can be used stably under circumstances in which the electrodes may be repeatedly bent for a certain period of time, which is typical for monitoring airborne biological agents continuously using roll-to-roll sensors. For this test, these electrodes were first prepared in a planar form and then bent by 90° before obtaining electrochemical measurements using DPV for each of two states, i.e., planar and bent. For each bending-and-unfolding cycle, about 5 μl of deionized (DI) water was applied to prevent dehydration of the sensing areas and to maintain the ion concentration of the electrolyte for stable electrochemical measurements; these measurement cycles were repeated twenty times.

### Electrochemical measurements

The electrochemical measurements were conducted at room temperature using Autolab PGSTAT204 and NOVA 1.10 software (Metrohm, Netherlands) using a mixture of 10 mM ferri/ferrocyanide and 0.5 M KCl in 1 × PBS solution as a redox mediator. Graph analysis was conducted using Origin 2017. Changes in the electrochemical properties of the MWCNT-PDMS, COOH-CNT/MWCNT-PDMS, Ab/COOH-CNT/MWCNT-PDMS, and BSA/Ab/COOH-CNT/MWCNT-PDMS electrodes were measured using cyclic voltammetry (CV), DPV, and electrochemical impedance spectroscopy (EIS). Optimization and HA protein detection experiments, and selectivity and reproducibility tests were performed using DPV.
